# Bis[4-bromo-2-(cyclo­pentyl­imino­meth­yl)phenolato]copper(II)

**DOI:** 10.1107/S1600536809006606

**Published:** 2009-02-28

**Authors:** Bang-Hong Cai

**Affiliations:** a Department of Chemistry, Jiaying University, Meizhou Guangdong 514015, People’s Republic of China

## Abstract

The title compound, [Cu(C_12_H_13_BrNO)_2_], was prepared by the reaction of 5-bromo­salicylaldehyde, cyclo­pentyl­amine and copper(II) acetate in an ethanol solution. The Cu^II^ atom lies on an inversion center and is four-coordinated in a square-planar geometry by two N and two O atoms from two 4-bromo-2-(cyclo­pentyl­imino­meth­yl)phenolate Schiff base ligands.

## Related literature

For background on Schiff base complexes, see: Costes *et al.* (2002 ▶); Erxleben (2001 ▶); Lacroix *et al.* (1996 ▶); Odoko *et al.* (2006 ▶); Ali *et al.* (2006 ▶). For related copper(II) complexes, see: Wang *et al.* (2007 ▶); Datta *et al.* (2008 ▶); Yusnita *et al.* (2008 ▶); Wang & Zheng (2007 ▶). For a related zinc(II) complex, see: Cai (2009 ▶).
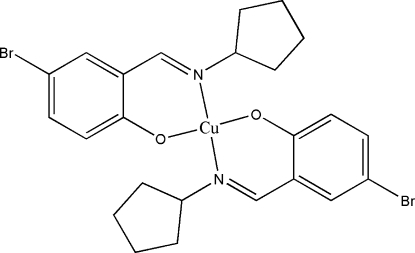


## Experimental

### 

#### Crystal data

[Cu(C_12_H_13_BrNO)_2_]
*M* *_r_* = 597.83
Monoclinic, 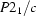

*a* = 9.190 (2) Å
*b* = 10.960 (2) Å
*c* = 12.166 (2) Å
β = 109.73 (3)°
*V* = 1153.5 (4) Å^3^
*Z* = 2
Mo *K*α radiation
μ = 4.44 mm^−1^
*T* = 298 K
0.27 × 0.23 × 0.23 mm

#### Data collection

Bruker SMART 1000 CCD area-detector diffractometer
Absorption correction: multi-scan (*SADABS*; Sheldrick, 1996 ▶) *T*_min_ = 0.381, *T*_max_ = 0.429 (expected range = 0.320–0.361)
9709 measured reflections
2636 independent reflections
2003 reflections with *I* > 2σ(*I*)
*R*_int_ = 0.035

#### Refinement

*R*[*F* ^2^ > 2σ(*F* ^2^)] = 0.039
*wR*(*F* ^2^) = 0.098
*S* = 1.02
2636 reflections
142 parameters
H-atom parameters constrained
Δρ_max_ = 0.93 e Å^−3^
Δρ_min_ = −0.42 e Å^−3^

### 

Data collection: *SMART* (Bruker, 2002 ▶); cell refinement: *SAINT* (Bruker, 2002 ▶); data reduction: *SAINT*; program(s) used to solve structure: *SHELXS97* (Sheldrick, 2008 ▶); program(s) used to refine structure: *SHELXL97* (Sheldrick, 2008 ▶); molecular graphics: *SHELXTL* (Sheldrick, 2008 ▶); software used to prepare material for publication: *SHELXTL*.

## Supplementary Material

Crystal structure: contains datablocks global, I. DOI: 10.1107/S1600536809006606/su2099sup1.cif

Structure factors: contains datablocks I. DOI: 10.1107/S1600536809006606/su2099Isup2.hkl

Additional supplementary materials:  crystallographic information; 3D view; checkCIF report

## Figures and Tables

**Fig. 1.**
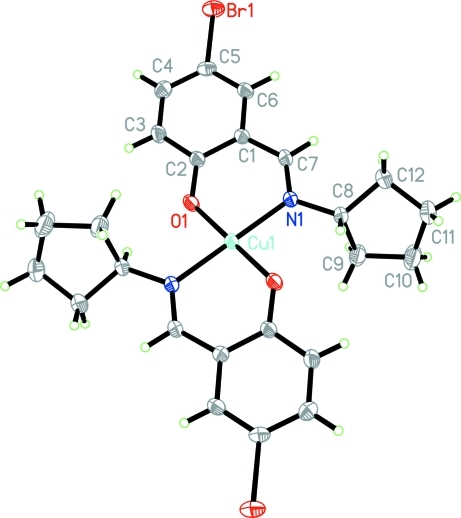
The molecular structure of the title compound, showing 30% probability displacement ellipsoids [unlabelled atoms are related to the labelled atoms by the symmetry operation -x, 2-y, -z].

## supplementary crystallographic information

### Comment

Schiff bases are interesting ligands which form a large number of complexes with metal atoms (Costes *et al.*, 2002; Erxleben, 2001; Lacroix *et al.*, 1996; Odoko *et al.*, 2006; Ali *et al.*, 2006). The author has recently reported on the crystal structure of a zinc(II) complex with the Schiff base (2-morpholin-4-ylethyl)-(1-pyridin-2-ylmethylidene)amine (Cai, 2009). As a continuous of our work in this area, we report here on the crystal structure of the title copper(II) complex (Fig. 1), derived from the Schiff base 4-bromo-2-(cyclopentyliminomethyl)phenol.

In the centrosymmetric title complex the Cu^II^ atom, is located on an inversion center, and is four-coordinate in a square planar geometry with two nitrogen and two oxygen atoms from two Schiff base ligands. All the coordinate bond lengths are typical and comparable with those in similar copper(II) complexes (Wang *et al.*, 2007; Datta *et al.*, 2008; Yusnita *et al.*, 2008; Wang & Zheng, 2007).

### Experimental

5-Bromosalicylaldehyde (0.2 mmol, 40.3 mg), cyclopentylamine (0.2 mmol, 17.0 mg), and copper(II) acetate monohydrate (0.1 mmol, 20.0 mg) were mixed in 20 ml of ethanol. The mixture was stirred for 2 h at rt, giving a blue solution. Single-crystals were formed by gradual evaporation of the solution in air after several days.

### Refinement

H atoms were placed in calculated positions and treated as riding: C–H = 0.93–0.98 Å, with *U*_iso_(H) = 1.2*U*_eq_(C).

### Crystal data

**Table:**

[Cu(C_12_H_13_BrNO)_2_]	*F*(000) = 598
*M**_r_* = 597.83	*D*_x_ = 1.721 Mg m^−^^3^
Monoclinic, *P*2_1_/*c*	Mo *K*α radiation, λ = 0.71073 Å
*a* = 9.190 (2) Å	Cell parameters from 2505 reflections
*b* = 10.960 (2) Å	θ = 2.3–25.0°
*c* = 12.166 (2) Å	µ = 4.43 mm^−^^1^
β = 109.73 (3)°	*T* = 298 K
*V* = 1153.5 (4) Å^3^	Block, blue
*Z* = 2	0.27 × 0.23 × 0.23 mm

### Data collection

**Table:**

Bruker SMART 1000 CCD area-detector diffractometer	2636 independent reflections
Radiation source: fine-focus sealed tube	2003 reflections with *I* > 2σ(*I*)
graphite	*R*_int_ = 0.035
ω scans	θ_max_ = 27.5°, θ_min_ = 2.4°
Absorption correction: multi-scan (*SADABS*; Sheldrick, 1996)	*h* = −11→11
*T*_min_ = 0.381, *T*_max_ = 0.429	*k* = −14→14
9709 measured reflections	*l* = −15→15

### Refinement

**Table:**

Refinement on *F*^2^	Primary atom site location: structure-invariant direct methods
Least-squares matrix: full	Secondary atom site location: difference Fourier map
*R*[*F*^2^ > 2σ(*F*^2^)] = 0.039	Hydrogen site location: inferred from neighbouring sites
*wR*(*F*^2^) = 0.098	H-atom parameters constrained
*S* = 1.02	*w* = 1/[σ^2^(*F*_o_^2^) + (0.0427*P*)^2^ + 0.9221*P*] where *P* = (*F*_o_^2^ + 2*F*_c_^2^)/3
2636 reflections	(Δ/σ)_max_ = 0.001
142 parameters	Δρ_max_ = 0.93 e Å^−^^3^
0 restraints	Δρ_min_ = −0.41 e Å^−^^3^

### Special details

**Table:**

Geometry. All esds (except the esd in the dihedral angle between two l.s. planes) are estimated using the full covariance matrix. The cell esds are taken into account individually in the estimation of esds in distances, angles and torsion angles; correlations between esds in cell parameters are only used when they are defined by crystal symmetry. An approximate (isotropic) treatment of cell esds is used for estimating esds involving l.s. planes.
Refinement. Refinement of F^2^ against ALL reflections. The weighted R-factor wR and goodness of fit S are based on F^2^, conventional R-factors R are based on F, with F set to zero for negative F^2^. The threshold expression of F^2^ > 2sigma(F^2^) is used only for calculating R-factors(gt) etc. and is not relevant to the choice of reflections for refinement. R-factors based on F^2^ are statistically about twice as large as those based on F, and R- factors based on ALL data will be even larger.

### Fractional atomic coordinates and isotropic or equivalent isotropic displacement parameters (Å^2^)

**Table:**

	*x*	*y*	*z*	*U*_iso_*/*U*_eq_	
Cu1	0.0000	1.0000	0.0000	0.03400 (16)	
Br1	−0.34783 (5)	0.60773 (4)	0.33661 (4)	0.06123 (17)	
O1	−0.0883 (3)	1.00623 (19)	0.1197 (2)	0.0446 (6)	
N1	0.0534 (3)	0.8203 (2)	0.0328 (2)	0.0341 (6)	
C1	−0.1071 (4)	0.7941 (3)	0.1548 (3)	0.0344 (7)	
C2	−0.1456 (4)	0.9175 (3)	0.1630 (3)	0.0348 (7)	
C3	−0.2475 (4)	0.9435 (3)	0.2236 (3)	0.0435 (8)	
H3	−0.2753	1.0242	0.2294	0.052*	
C4	−0.3073 (4)	0.8541 (3)	0.2744 (3)	0.0465 (9)	
H4	−0.3752	0.8741	0.3136	0.056*	
C5	−0.2659 (4)	0.7337 (3)	0.2671 (3)	0.0422 (8)	
C6	−0.1676 (4)	0.7034 (3)	0.2090 (3)	0.0391 (7)	
H6	−0.1404	0.6222	0.2052	0.047*	
C7	−0.0063 (4)	0.7551 (3)	0.0935 (3)	0.0374 (7)	
H7	0.0184	0.6726	0.0987	0.045*	
C8	0.1560 (4)	0.7602 (3)	−0.0219 (3)	0.0386 (7)	
H8	0.1265	0.7915	−0.1019	0.046*	
C9	0.3252 (4)	0.7923 (4)	0.0374 (4)	0.0603 (11)	
H9A	0.3467	0.8740	0.0163	0.072*	
H9B	0.3536	0.7874	0.1216	0.072*	
C10	0.4115 (5)	0.6976 (5)	−0.0078 (6)	0.0901 (18)	
H10A	0.4543	0.7346	−0.0626	0.108*	
H10B	0.4955	0.6632	0.0563	0.108*	
C11	0.2977 (5)	0.6002 (4)	−0.0671 (4)	0.0642 (12)	
H11A	0.3412	0.5199	−0.0429	0.077*	
H11B	0.2696	0.6065	−0.1512	0.077*	
C12	0.1586 (5)	0.6206 (3)	−0.0307 (4)	0.0507 (9)	
H12A	0.1713	0.5824	0.0438	0.061*	
H12B	0.0651	0.5900	−0.0889	0.061*	

### Atomic displacement parameters (Å^2^)

**Table:**

	*U*^11^	*U*^22^	*U*^33^	*U*^12^	*U*^13^	*U*^23^
Cu1	0.0412 (3)	0.0242 (3)	0.0424 (3)	0.0024 (2)	0.0218 (3)	0.0028 (2)
Br1	0.0702 (3)	0.0547 (3)	0.0720 (3)	−0.0114 (2)	0.0414 (2)	0.0125 (2)
O1	0.0659 (16)	0.0251 (11)	0.0570 (15)	0.0009 (11)	0.0395 (13)	0.0012 (10)
N1	0.0374 (14)	0.0263 (13)	0.0425 (15)	0.0034 (11)	0.0185 (12)	0.0018 (11)
C1	0.0377 (17)	0.0294 (16)	0.0387 (17)	0.0018 (13)	0.0163 (14)	0.0037 (13)
C2	0.0401 (18)	0.0290 (15)	0.0373 (17)	0.0023 (13)	0.0159 (14)	0.0031 (13)
C3	0.056 (2)	0.0336 (17)	0.050 (2)	0.0058 (16)	0.0300 (18)	−0.0006 (16)
C4	0.050 (2)	0.049 (2)	0.049 (2)	0.0019 (17)	0.0287 (18)	0.0009 (17)
C5	0.0443 (19)	0.0424 (19)	0.0442 (19)	−0.0079 (15)	0.0205 (16)	0.0070 (15)
C6	0.0461 (19)	0.0289 (16)	0.0441 (19)	−0.0008 (14)	0.0175 (15)	0.0036 (14)
C7	0.0406 (17)	0.0270 (16)	0.0470 (19)	0.0064 (13)	0.0177 (15)	0.0040 (14)
C8	0.0421 (18)	0.0292 (16)	0.0489 (19)	0.0060 (14)	0.0212 (16)	0.0012 (14)
C9	0.043 (2)	0.056 (2)	0.085 (3)	−0.0012 (19)	0.026 (2)	−0.024 (2)
C10	0.051 (3)	0.082 (3)	0.147 (5)	−0.006 (2)	0.046 (3)	−0.051 (4)
C11	0.066 (3)	0.049 (2)	0.085 (3)	0.012 (2)	0.036 (2)	−0.010 (2)
C12	0.059 (2)	0.0349 (19)	0.064 (2)	0.0030 (17)	0.028 (2)	−0.0058 (17)

### Geometric parameters (Å, °)

**Table:**

Cu1—O1^i^	1.892 (2)	C6—H6	0.9300
Cu1—O1	1.892 (2)	C7—H7	0.9300
Cu1—N1	2.036 (2)	C8—C9	1.518 (5)
Cu1—N1^i^	2.036 (2)	C8—C12	1.534 (4)
Br1—C5	1.902 (3)	C8—H8	0.9800
O1—C2	1.299 (4)	C9—C10	1.518 (5)
N1—C7	1.278 (4)	C9—H9A	0.9700
N1—C8	1.480 (4)	C9—H9B	0.9700
C1—C6	1.407 (4)	C10—C11	1.498 (6)
C1—C2	1.411 (4)	C10—H10A	0.9700
C1—C7	1.436 (4)	C10—H10B	0.9700
C2—C3	1.403 (4)	C11—C12	1.504 (6)
C3—C4	1.369 (5)	C11—H11A	0.9700
C3—H3	0.9300	C11—H11B	0.9700
C4—C5	1.385 (5)	C12—H12A	0.9700
C4—H4	0.9300	C12—H12B	0.9700
C5—C6	1.362 (5)		
			
O1^i^—Cu1—O1	180.0	N1—C8—C9	112.9 (3)
O1^i^—Cu1—N1	88.74 (10)	N1—C8—C12	120.2 (3)
O1—Cu1—N1	91.26 (10)	C9—C8—C12	103.1 (3)
O1^i^—Cu1—N1^i^	91.26 (10)	N1—C8—H8	106.6
O1—Cu1—N1^i^	88.74 (10)	C9—C8—H8	106.6
N1—Cu1—N1^i^	180.0	C12—C8—H8	106.6
C2—O1—Cu1	128.5 (2)	C10—C9—C8	104.2 (3)
C7—N1—C8	118.3 (3)	C10—C9—H9A	110.9
C7—N1—Cu1	122.1 (2)	C8—C9—H9A	110.9
C8—N1—Cu1	119.42 (19)	C10—C9—H9B	110.9
C6—C1—C2	119.7 (3)	C8—C9—H9B	110.9
C6—C1—C7	117.4 (3)	H9A—C9—H9B	108.9
C2—C1—C7	122.9 (3)	C11—C10—C9	107.3 (3)
O1—C2—C3	119.7 (3)	C11—C10—H10A	110.3
O1—C2—C1	122.9 (3)	C9—C10—H10A	110.3
C3—C2—C1	117.3 (3)	C11—C10—H10B	110.3
C4—C3—C2	122.3 (3)	C9—C10—H10B	110.3
C4—C3—H3	118.9	H10A—C10—H10B	108.5
C2—C3—H3	118.9	C10—C11—C12	106.1 (3)
C3—C4—C5	119.5 (3)	C10—C11—H11A	110.5
C3—C4—H4	120.3	C12—C11—H11A	110.5
C5—C4—H4	120.3	C10—C11—H11B	110.5
C6—C5—C4	120.7 (3)	C12—C11—H11B	110.5
C6—C5—Br1	119.0 (3)	H11A—C11—H11B	108.7
C4—C5—Br1	120.3 (3)	C11—C12—C8	101.8 (3)
C5—C6—C1	120.5 (3)	C11—C12—H12A	111.4
C5—C6—H6	119.7	C8—C12—H12A	111.4
C1—C6—H6	119.7	C11—C12—H12B	111.4
N1—C7—C1	127.8 (3)	C8—C12—H12B	111.4
N1—C7—H7	116.1	H12A—C12—H12B	109.3
C1—C7—H7	116.1		

Symmetry codes: (i) −*x*, −*y*+2, −*z*.
